# Supplementary material for: Mutual induced-fit mechanism drives binding between intrinsically disordered Bim and cryptic binding site of Bcl-xL
Source: Commun Biol. 2023 Mar 30;6:349. doi: 10.1038/s42003-023-04720-6 (PMC10063584; doi:10.1038/s42003-023-04720-6)
Supplement: Supplementary file 2 — Supplementary Information [file 42003_2023_4720_MOESM2_ESM.pdf]

Mutual Induced-Fit Mechanism Drives Binding Between Intrinsically Disordered Bim and Cryptic Binding Site of Bcl-xL

Gert-Jan Bekker[1,*], **Mitsugu Araki**[2], **Kanji Oshima**[3], **Yasushi Okuno**[2], and **Narutoshi Kamiya**[4,*]

[1] Institute for Protein Research, Osaka University, 3-2 Yamadaoka, Suita, Osaka 565-0871, Japan
[2] Graduate School of Medicine, Kyoto University, 53 Shogoin-Kawaharacho, Sakyo-ku, Kyoto. 606-8507, Japan.
[3] Bio-Pharma Research Laboratories, KANEKA CORPORATION, 1-8 Miyamae-cho, Takasago-cho, Takasago, Hyogo 676-8688, Japan.
[4] Graduate School of Information Science, University of Hyogo, 7-1-28 Minatojima Minami-machi, Chuo-ku, Kobe, Hyogo 650-0047, Japan.

[*]Correspondence and requests for materials should be addressed to G.J.B. (email: gertjan.bekker@protein.osaka-u.ac.jp) or N.K. (email: n.kamiya@sim.u-hyogo.ac.jp)

Table of contents

**Note S1**	Multicanonical MD algorithm	S3
**Fig. S1**	Overview of role of Bim and Bcl-xL in the apoptosis process	S4
**Fig. S2**	Multicanonical potential energy distributions of Bim in isolation using two forcefields	S5
**Fig. S3**	Free energy landscapes of Bim in isolation using two forcefields	S6
**Fig. S4**	Multicanonical potential energy distribution of Bcl-xL – Bim dynamic docking	S7
**Fig. S5**	3D structure of picked representative configurations $\boldsymbol{r}_k$ and the experimental structure	S8
**Fig. S6**	Crystal packing observed in the experimental structure	S10
**Fig. S7**	3D structure of picked representative configurations $\boldsymbol{r}_k$ and $\boldsymbol{q}_k$	S11
**Fig. S8**	Overview of binding pathway obtained from the multicanonical ensemble	S13
**Fig. S9**	Location of residues that form the distance pairs used for pocket size analysis	S14
**Fig. S10**	Reweighted distributions of distances of five pairs of residues	S15
**Fig. S11**	Comparison between Bcl-xL – Bim complex structures from r_1, r_6 and r_8	S16
**Fig. S12**	Comparison between Bim helix propensity between Bim in isolation and in the presence of Bcl-xL	S17
**Fig. S13**	Free energy landscapes of Bim in isolation using AMBER ff99SB-ILDN with OPC waters	S18
**Fig. S14**	Comparison of chemical structures.	S19
**Table S1**	Results from Bim sampling simulations (in isolation) with the AMBER ff14SB force field with TIP3P waters	S20
**Table S2**	Results from Bim sampling simulations (in isolation) with the AMBER ff99SB-ILDN force field with TIP3P waters	S22
**Table S3**	Convergence of McMD dynamic docking simulations	S25
**Table S4**	McMD-based dynamic docking results using subsets of the simulation data	S27
**Table S5**	Per-residue R-values of Bim during 400 K canonical simulations	S29
**Table S6**	Picking statistics from the McMD ensemble to produce the binding pathway for the path sampling simulations	S30
**Table S7**	Distance between Cα atoms of residue pairs in structures r_k and r_k^{bcl}	S31
**Table S8**	Results from Bim sampling simulations (in isolation) with the AMBER ff99SB-ILDN force field and the OPC water force field	S32
**Table S9**	System & simulation parameters for Bcl-xL – Bim binding simulations.	S36
	Supplementary References	S37

Note S1: Multicanonical MD algorithm

We used our own developed McMD-based dynamic docking method that has been thoroughly described in various previous papers,[1–9] but here we will shortly review the algorithm. The probability distribution of the potential energy of the multicanonical ensemble is defined by the following equation:

$$P_{mc}(E, T_0) = \frac{1}{Z_{mc}} n(E) e^{-W(E)} = constant \tag{S1}$$

where E is the potential energy, T_0 the simulation temperature, $n(E)$ the density of states and Z_{mc} the partition function:

$$Z_{mc} = \int n(E) e^{-W(E)} dE \tag{S2}$$

$W(E)$ is a weighting function to modulate the probability distribution P_{mc} in order for it to become constant and enables the system to take a random walk along the target energy range, and is defined as follows:

$$W(E) = \ln n(E) = \frac{E}{RT_0} + \ln P_c(E, T_0) \tag{S3}$$

where R is the gas constant and P_c the canonical energy distribution at T_0. During the McMD simulations, this weighting function is used to scale the forces by a factor of $\nabla W(E)$, where the multicanonical temperature T_{mc}, which corresponds to $T_0/\nabla W(E)$, is restricted to a specific target range between T_{low} to T_{high}, which we generally set at 280 K and 700 K, respectively. Multiple iterations of sampling are required to estimate the correct bias that enables a random walk along a wide energy range, where the weighting function is updated between iterations using:

$$W^{i+1}(E) = W^{i}(E) + \ln P^{i}_{mc}(E, T_0) \tag{S4}$$

After obtaining a flat potential energy distribution, a production run is executed to sample phase space. Due to the bias applied during the McMD simulations, the resulting multicanonical ensemble must be reweighted to obtain the canonical distribution at room temperature. A multicanonical distribution can be reweighted to a canonical distribution at any given temperature T within the flat energy range using the following equation:

$$P_c(E, T) = \frac{1}{Z_c} n(E) e^{-\frac{E}{RT}} = \frac{Z_{mc}}{Z_c} P_{mc}(E) e^{W(E)-\frac{E}{RT}} \tag{S5}$$

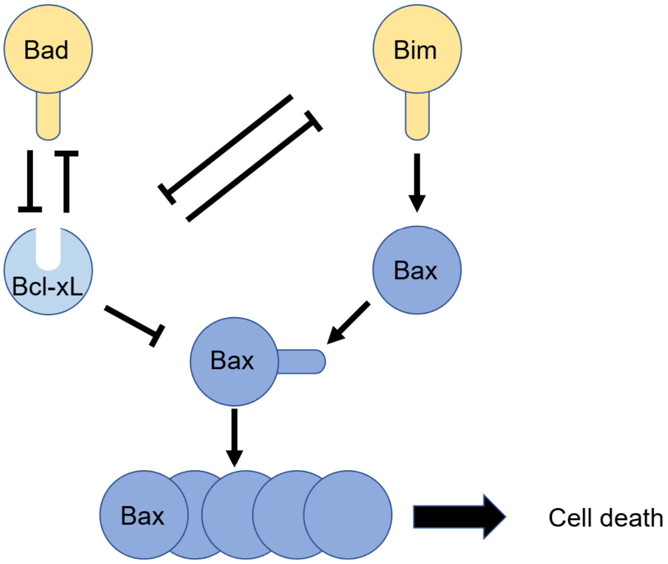

Fig. S1. Overview of role of Bim and Bcl-xL in the apoptosis process (adapted from Czabotar et al[10]).

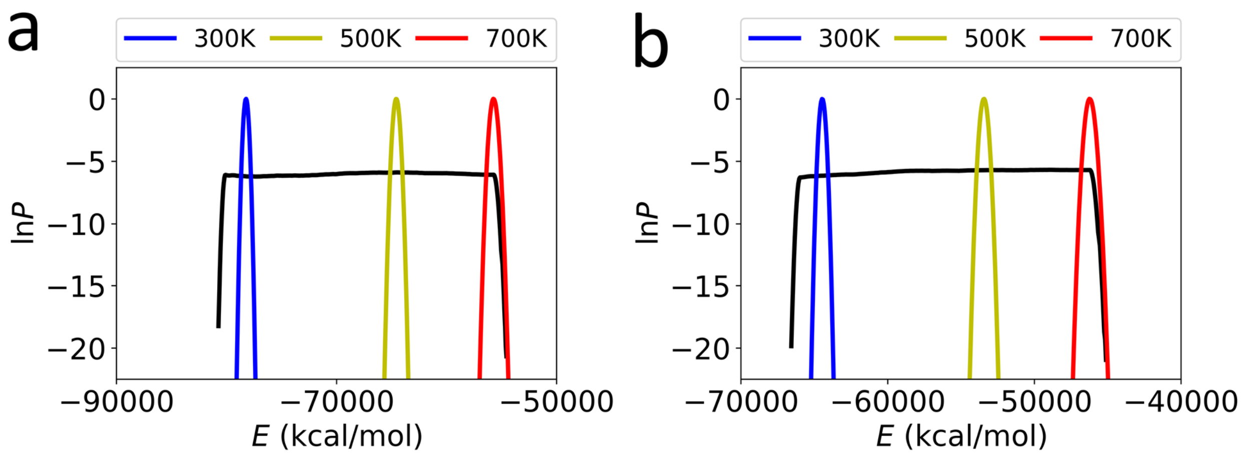

Fig. S2. Multicanonical potential energy distributions of Bim in isolation using two forcefields. Potential energy probability distribution ($P_{McMD}(E)$) as sampled during the production run. Also shown are the reweighing canonical distributions ($P_c(E, T)$) at 300 K, 500 K and 700 K in blue, yellow and red, respectively. a) Distribution obtained using the AMBER ff14SB forcefield. b) Distribution obtained using the AMBER ff99SB-ILDN forcefield.

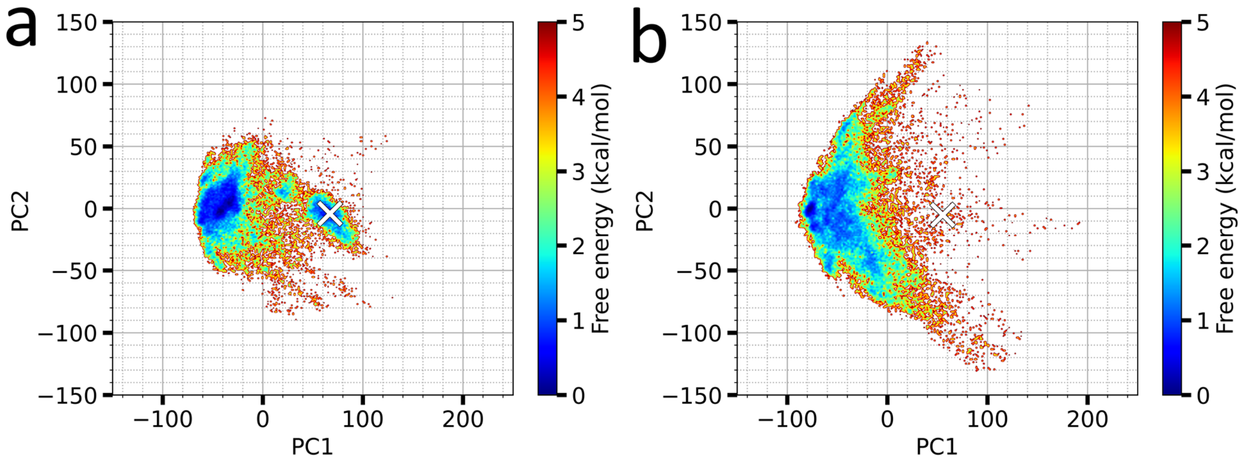

Fig. S3. Free energy landscapes of Bim in isolation using two forcefields. a) Free energy landscape obtained using the AMBER ff14SB forcefield. b) Free energy landscape obtained using the AMBER ff99SB-ILDN forcefield. In both cases, the X indicates the location of the experimental structure (Bim taken from PDB ID 4QVF). The PCAs were performed independently from each other.

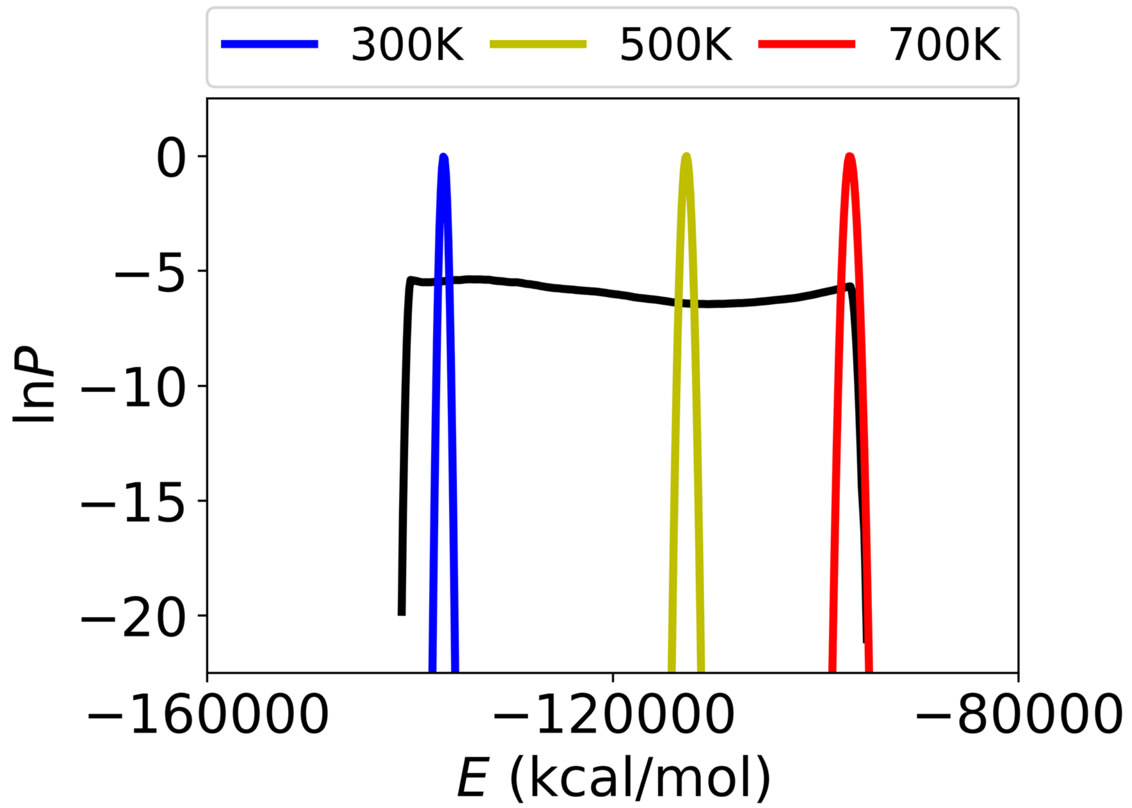

Fig. S4. Multicanonical potential energy distribution of Bcl-xL – Bim dynamic docking. Potential energy probability distribution ($P_{McMD}(E)$) as sampled during the production run. Also shown are the reweighing canonical distributions ($P_c(E, T)$) at 300 K, 500 K and 700 K in blue, yellow and red, respectively.

$\mathbf{r}_1$

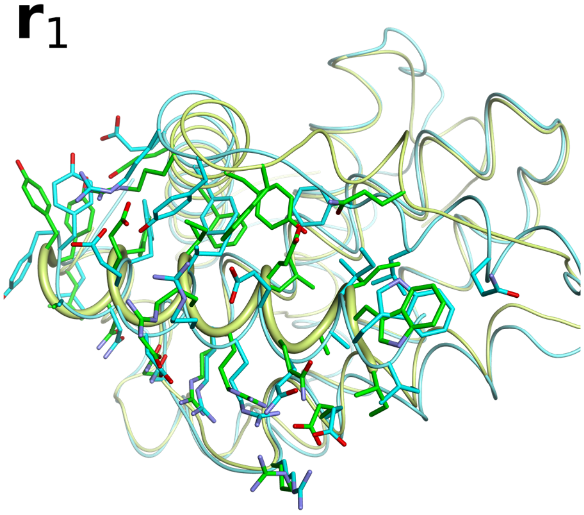

$\mathbf{r}_4$

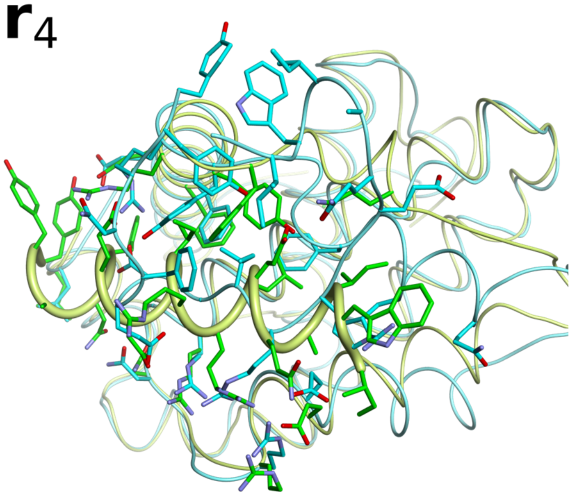

$\mathbf{r}_2$

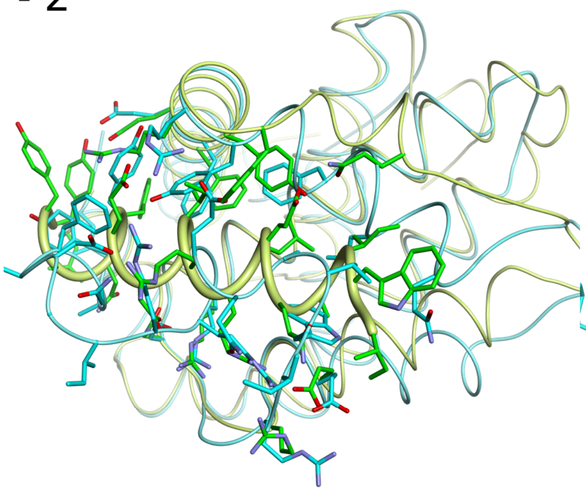

$\mathbf{r}_5$

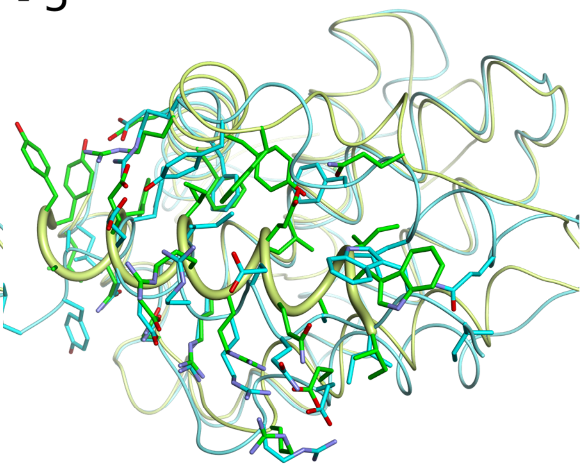

$\mathbf{r}_3$

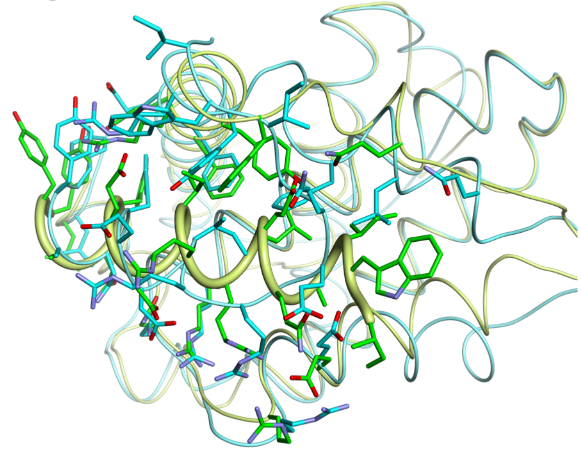

$\mathbf{r}_6$

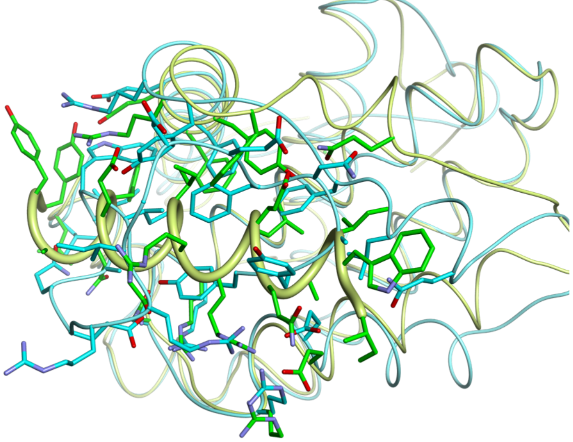

$\mathbf{r}_7$

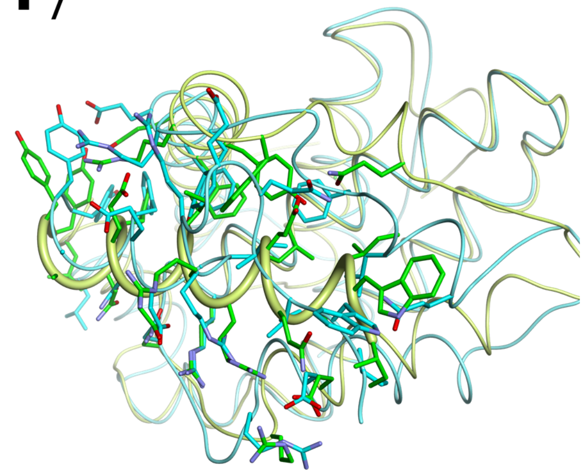

$\mathbf{r}_8$

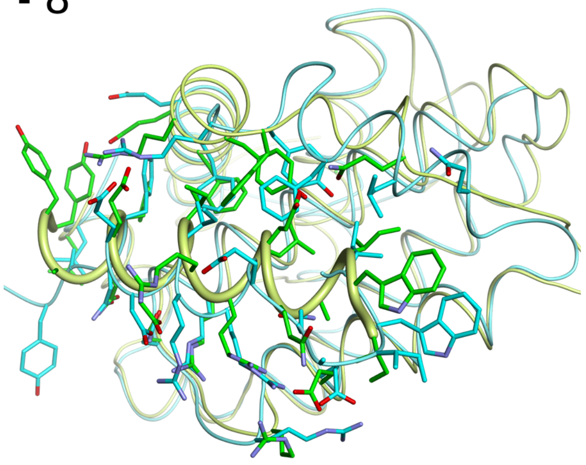

$\mathbf{r}_9$

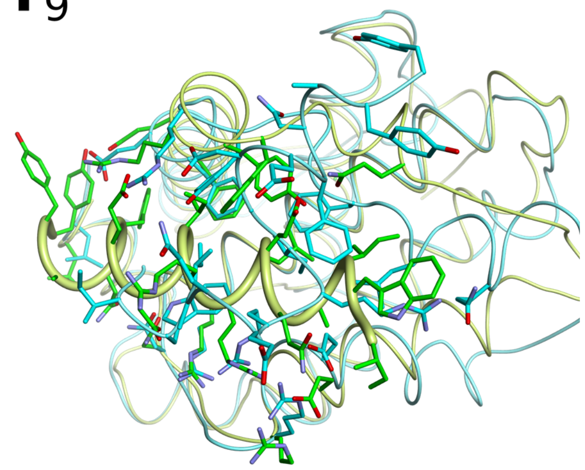

Fig. S5. 3D structure of picked representative configurations $\boldsymbol{r}_k$ and the experimental structure. Representative structures from the dynamic docking simulations $\boldsymbol{r}_k$ (cyan) and the experimental structure (green, PDB ID 4QVF) are shown with the sidechains of the nearby residues as their front view.

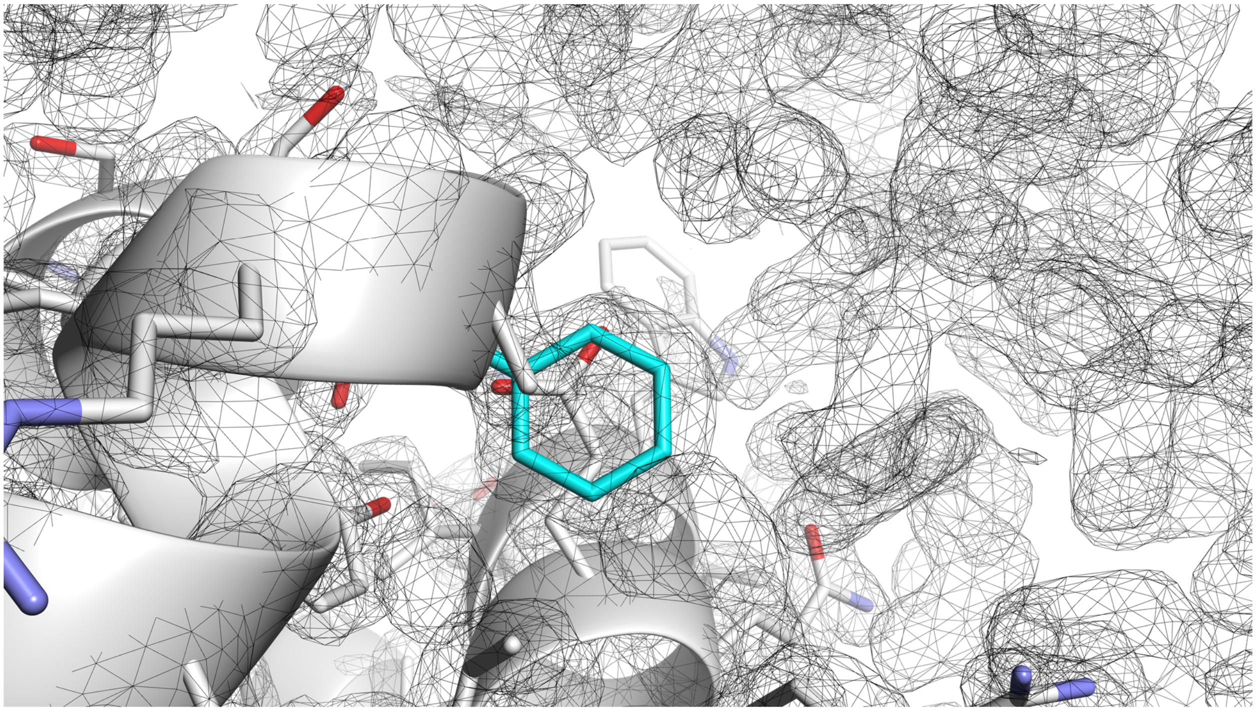

Fig. S6. Crystal packing observed in the experimental structure. Shown are the experimental structure (PDB ID 4QVF) around Phe105 (cyan) and the nearby crystal density.

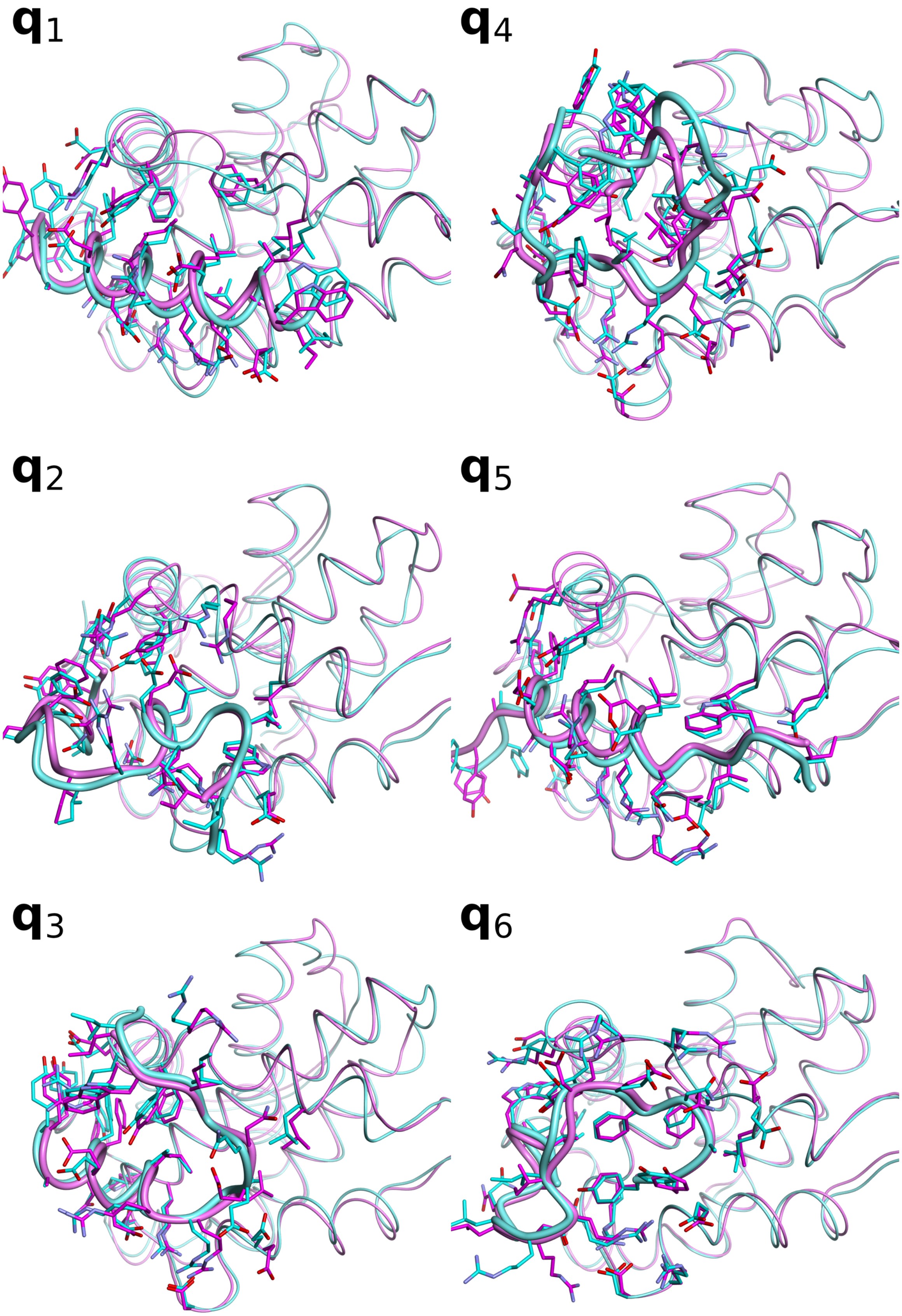

q_1
q_4
q_2
q_5
q_3
q_6

q7

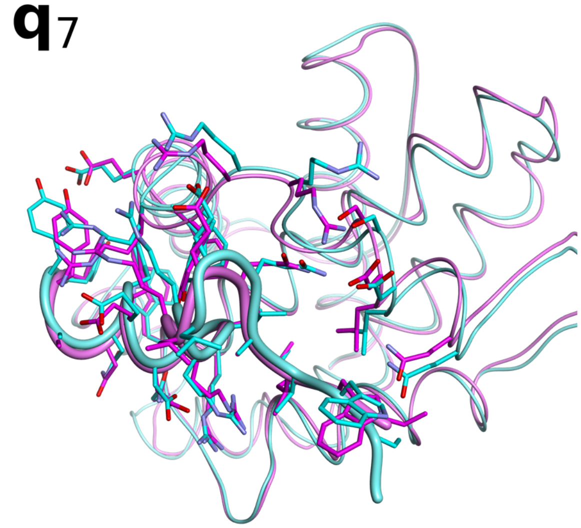

q8

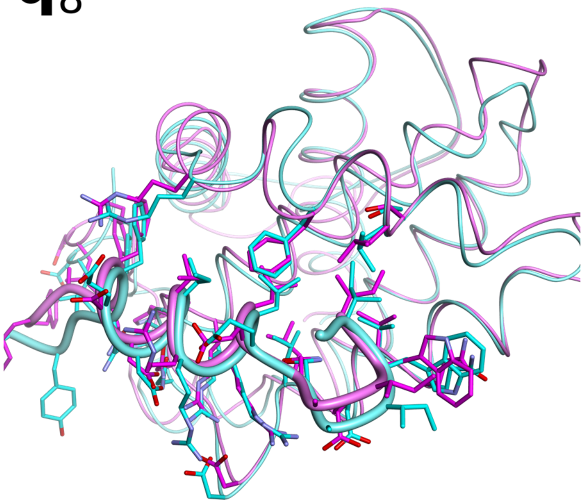

q9

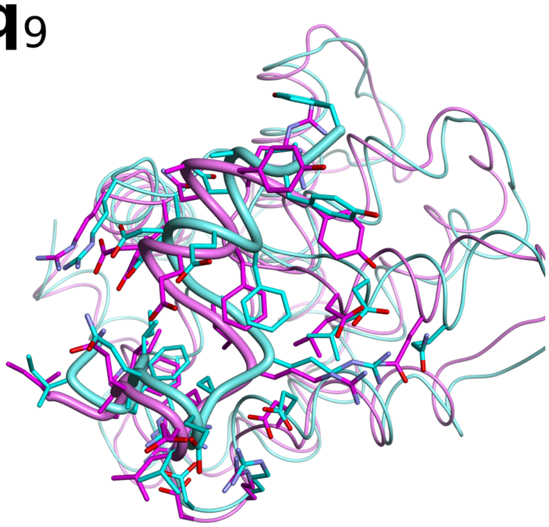

Fig. S7. 3D structure of picked representative configurations $\boldsymbol{r}_k$ and $\boldsymbol{q}_k$. Representative structures from the dynamic docking simulations $\boldsymbol{r}_k$ (cyan) and equilibrated structures $\boldsymbol{q}_k$ (magenta) are shown with the sidechains of the nearby residues are shown as their front view.

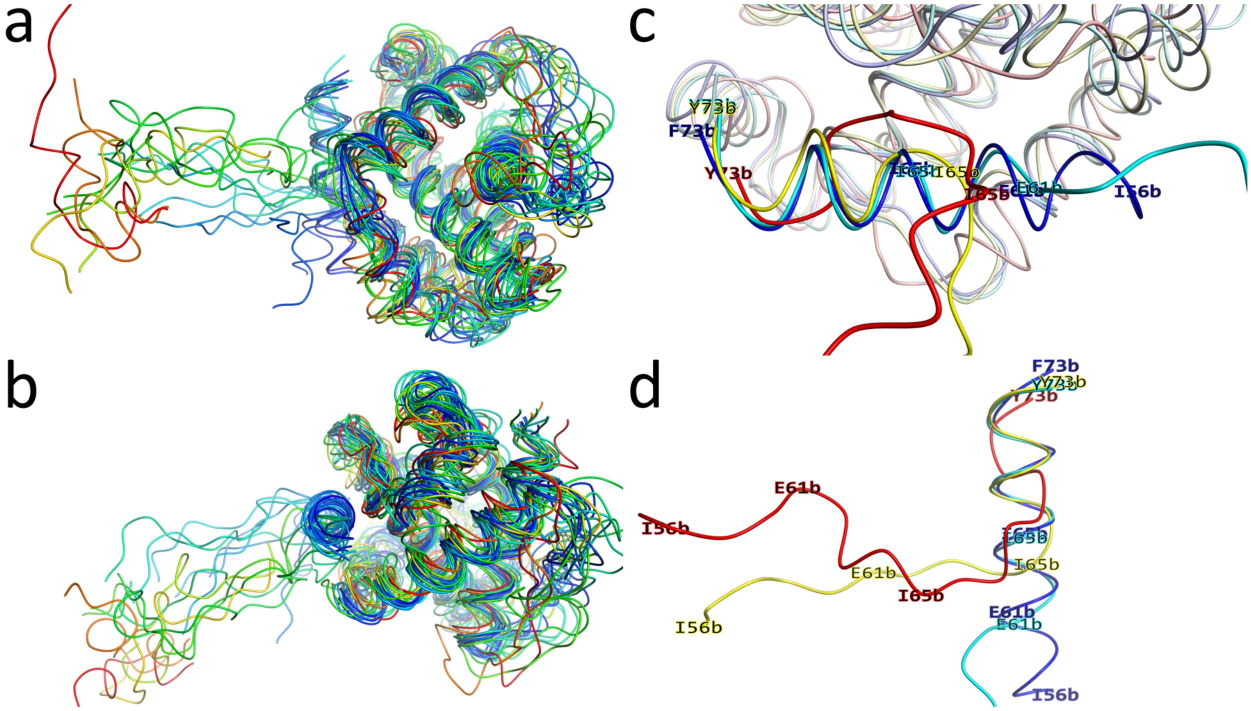

Fig. S8. Overview of binding pathway obtained from the multicanonical ensemble. a) Top view of 19 picked configurations along the binding/unbinding pathway, with Bcl-xL and Bim colored by their λ'-value from the bound state (λ' = 0 Å, corresponding to λ = 5.43 Å of $\mathbf{q}_1$, see Fig. 1c for a description of λ) to the unbound state (λ' = 36 Å) in a blue-red gradient. b) Side view of (a). c) Front view with only a subset of binding configurations. Shown are the structures at λ' values of 0 Å, 3 Å, 8 Å and 14 Å in blue, cyan, yellow and red, respectively. The structure of Bcl-xL is colored in the same way, but in a lighter shade than that used for Bim. Additionally, the residues Ile56b, Glu61b, Ile65b and Tyr73b are indicated. d) Top view of (c), but only showing Bim.

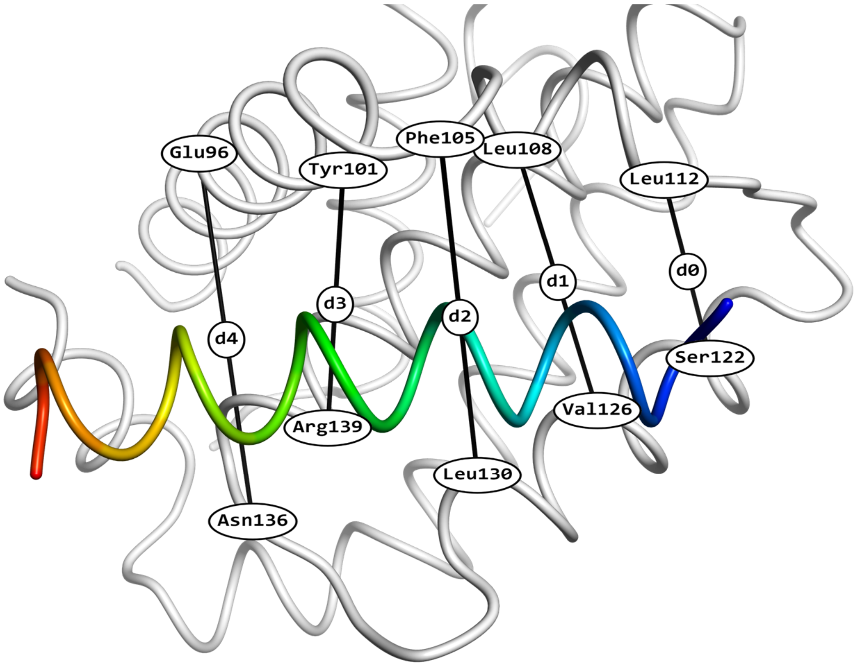

Fig. S9. Location of residues that form the distance pairs used for pocket size analysis. Shown is the holo complex structure between Bcl-xL (white) and Bim (blue-red gradient). Indicated are the Cα atoms of Leu112 – Ser122 (*d*0), Leu108 – Val126 (*d*1), Phe105 – Leu130 (*d*2), Tyr101 – Arg139 (*d*3) and Glu96 – Asn136 (*d*4) that form the distance pairs along the surface of the pocket where Bim binds.

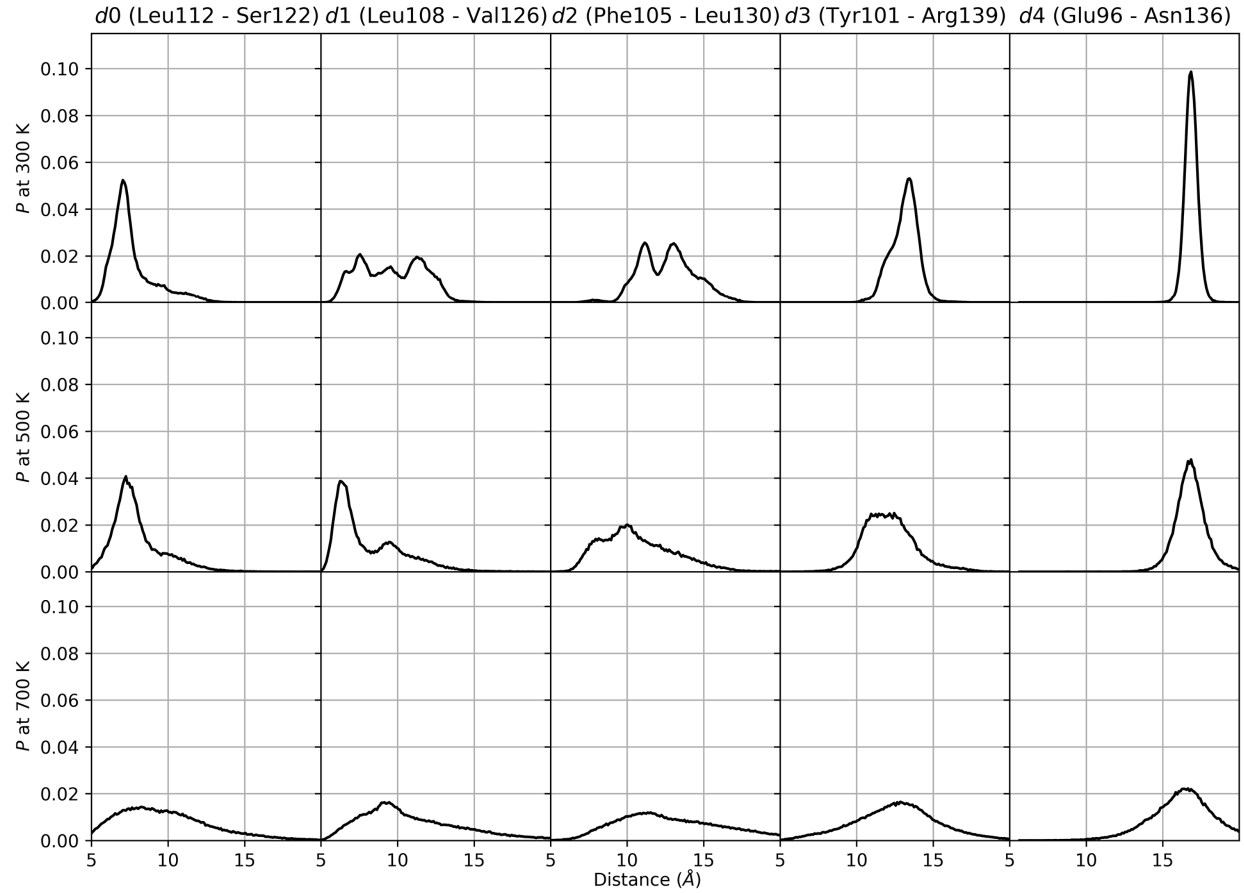

Fig. S10. Reweighted distributions of distances of five pairs of residues. Shown are the reweighted (300 K, 500 K and 700 K) probability distributions of distances between the Cα atoms of Leu112 – Ser122 (*d*0), Leu108 – Val126 (*d*1), Phe105 – Leu130 (*d*2), Tyr101 – Arg139 (*d*3) and Glu96 – Asn136 (*d*4). These pairs are along the surface of the binding site where Bim binds. The values corresponding to each of the structures $\mathbf{r}_k$ are shown in Table S5.

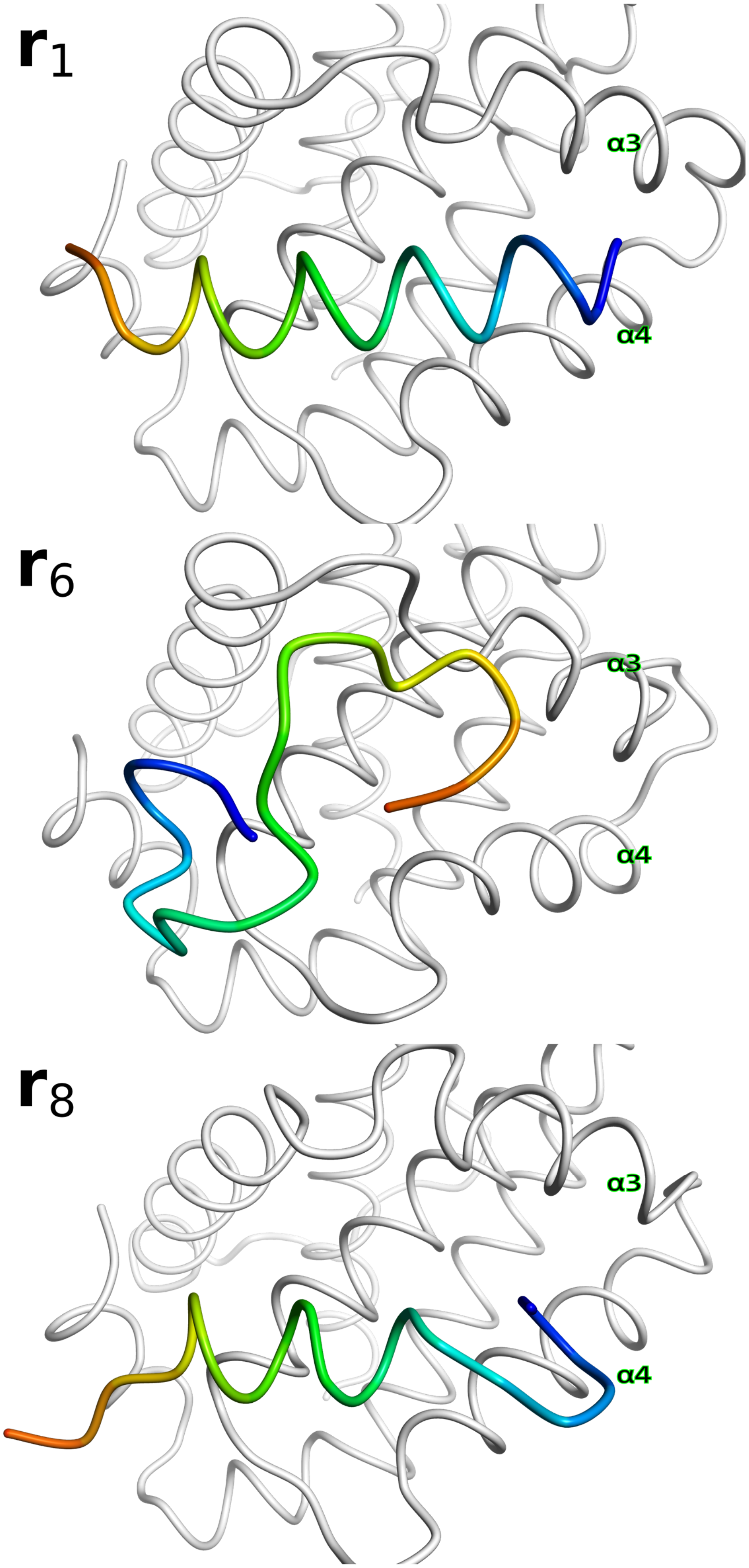

Fig. S11. Comparison between Bcl-xL – Bim complex structures from $\mathbf{r}_1$, $\mathbf{r}_6$ and $\mathbf{r}_8$. Shown are the structures of $\mathbf{r}_1$, $\mathbf{r}_6$ and $\mathbf{r}_8$, with Bcl-xL in white and Bim colored in a blue-red gradient. The values of the distances *d*0 – *d*4 are listed in Table S5.

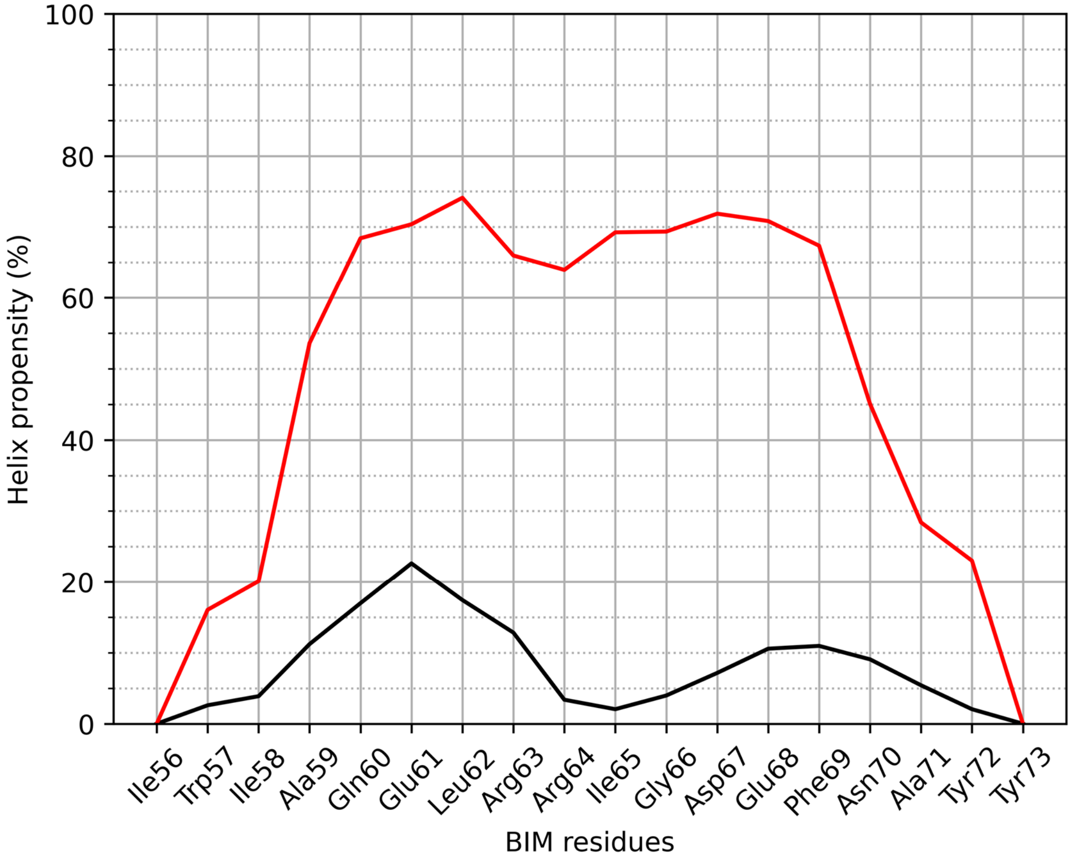

Fig. S12. Comparison between Bim helix propensity between Bim in isolation and in the presence of Bcl-xL. Reweighted (300 K) helical propensity calculated (by DSSP) for the ensemble obtained from our simulations of Bim in isolation (black) and Bim in the presence of Bcl-xL (red).

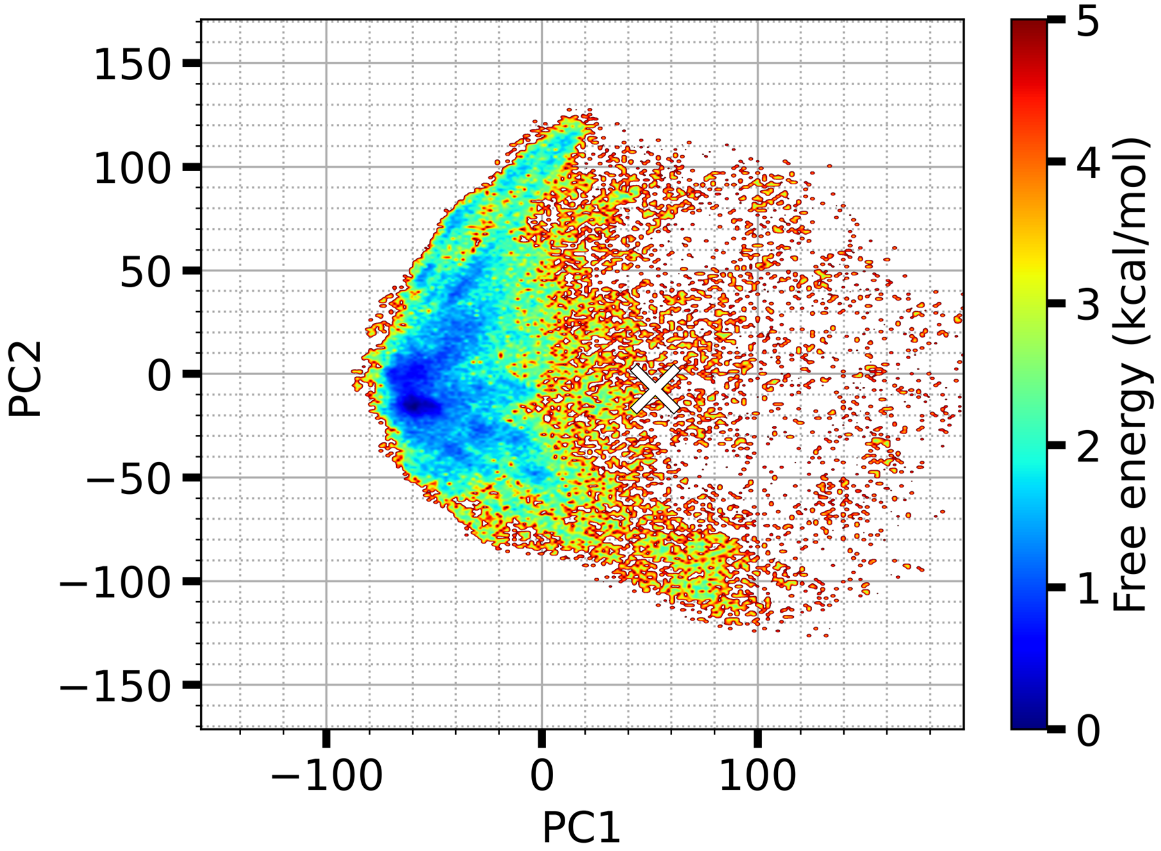

Fig. S13. Free energy landscape of Bim in isolation using AMBER ff99SB-ILDN with OPC waters. The X indicates the location of the experimental structure (Bim taken from PDB ID 4QVF). This PCA was performed independently from the PCAs of the simulations using TIP3P waters shown in Fig. S2.

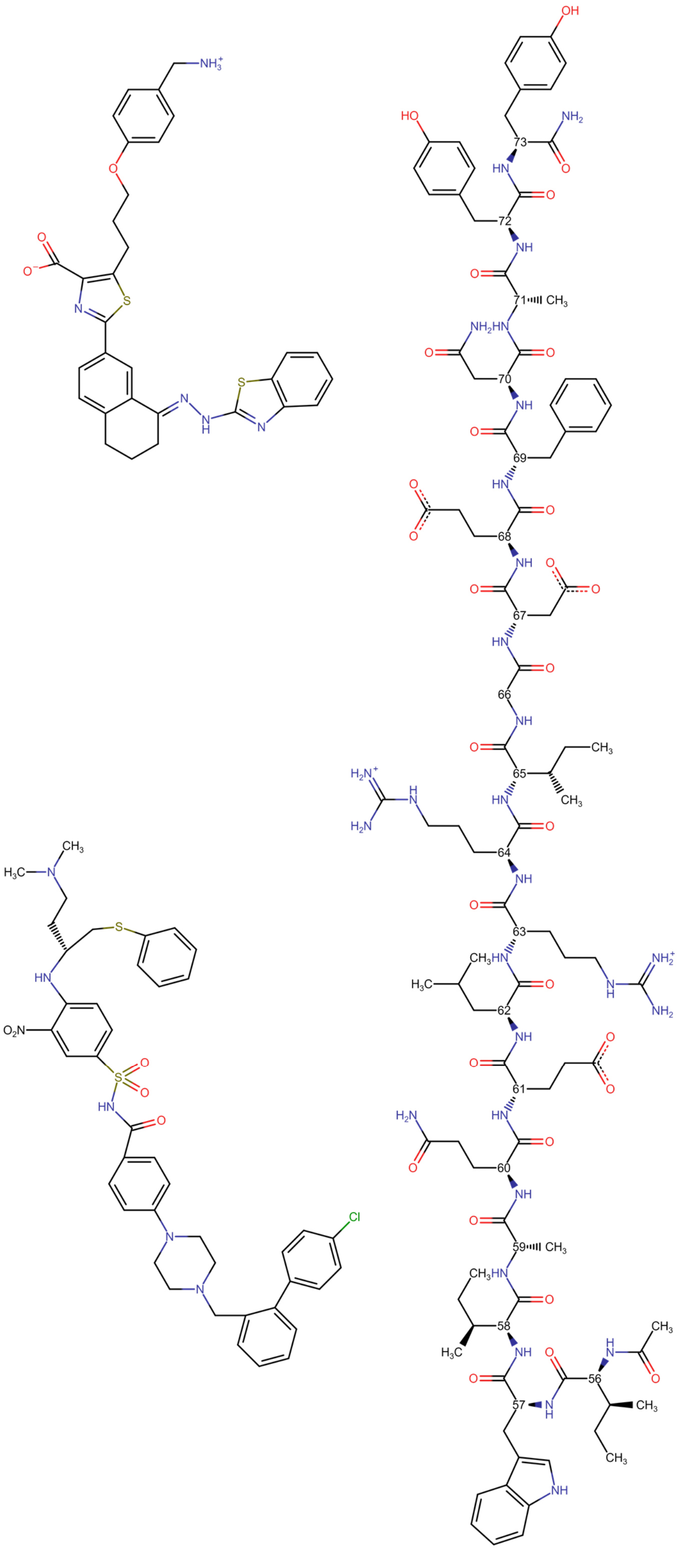

Fig. S14. Comparison of chemical structures. Chemical structures of WEHI-539 (top left), ABT-737 (bottom left) and Bim (right). For Bim, the residue number of each amino acid is placed on the location of the Cα in the figure.

Table S1. Results from Bim sampling simulations (in isolation) with the AMBER ff14SB force field with TIP3P waters. [a]

struc. Id	Cluster free energy	Population %	PC1	PC2	RASA	R(native)-value	RMSD
**r1**	0.00	16.14	65.30	-7.26	22.48	0.814	3.15
**r2**	0.08	14.00	-41.99	-0.79	19.41	0.617	7.56
**r3**	0.29	9.95	-26.69	9.32	22.74	0.236	21.26
**r4**	0.37	8.63	-56.53	-7.73	18.34	0.686	8.39
**r5**	0.68	5.19	-53.04	8.56	23.17	0.770	19.68
**r6**	0.78	4.34	-28.17	19.44	30.39	0.464	31.41
**r7**	1.19	2.20	-51.77	6.85	22.19	0.507	16.72
**r8**	1.30	1.81	10.46	-32.95	20.74	0.400	6.98
**r9**	1.32	1.76	-44.16	31.20	23.69	0.713	14.21
**r10**	1.45	1.41	-37.30	5.99	33.78	0.559	33.17
**r11**	1.53	1.24	-45.67	-22.29	22.99	0.759	13.90
**r12**	1.57	1.15	22.36	13.98	21.89	0.729	13.68
**r13**	1.59	1.12	-51.67	-33.22	28.30	0.370	30.05
**r14**	1.60	1.11	-36.79	14.40	18.65	0.840	8.06
**r15**	1.66	1.00	-59.11	26.83	18.55	0.175	9.74
**r16**	1.69	0.94	-27.61	-21.06	25.24	0.379	23.91
**r17**	1.73	0.89	-48.15	17.39	27.37	0.627	29.82
**r18**	1.74	0.87	-56.99	3.95	22.53	0.545	20.30
**r19**	1.80	0.78	-18.74	48.08	19.94	0.690	6.97
**r20**	1.84	0.74	-50.30	-17.37	22.72	0.525	17.07
**r21**	1.89	0.67	-47.31	7.44	23.60	0.618	22.37
**r22**	1.92	0.65	-26.83	20.48	28.45	0.604	32.14
**r23**	1.94	0.62	-39.09	6.14	29.95	0.495	32.46
**r24**	1.94	0.62	-0.91	-32.8	20.55	0.247	8.58
**r25**	1.96	0.60	-25.10	-2.35	24.16	0.763	20.24
**r26**	1.97	0.59	-22.26	-13.58	23.61	0.701	21.43
**r27**	2.02	0.54	-40.67	0.38	19.82	0.447	8.73
**r28**	2.04	0.52	45.22	-15.79	24.89	0.581	14.20
**r29**	2.08	0.50	-42.45	9.11	18.77	0.404	8.72
**r30**	2.08	0.49	-41.44	-10.64	32.56	0.570	31.76
**r31**	2.11	0.47	-49.82	12.03	20.39	0.567	8.78
**r32**	2.13	0.45	15.66	12.01	21.52	0.498	6.44
**r33**	2.14	0.45	-60.08	-0.39	22.60	0.666	17.49
**r34**	2.16	0.43	-6.43	17.03	20.40	0.570	7.09
**r35**	2.17	0.42	-43.78	18.62	30.24	0.658	30.72
**r36**	2.21	0.40	19.84	12.48	21.46	0.440	6.32
**r37**	2.23	0.38	-59.08	6.95	31.86	0.447	33.99
**r38**	2.25	0.37	-5.98	-1.55	20.04	0.838	7.96

**r39**	2.25	0.37	-3.13	45.56	20.05	0.753	6.28
**r40**	2.27	0.36	-29.46	-1.39	28.44	0.462	29.67
**r41**	2.27	0.36	-36.52	5.80	24.24	0.441	20.56
**r42**	2.29	0.35	7.64	-24.37	24.91	0.388	24.57
**r43**	2.30	0.34	-12.65	-9.20	19.26	0.592	8.18
**r44**	2.34	0.32	-30.60	-27.44	28.62	0.458	30.80
**r45**	2.35	0.31	18.31	-12.87	30.50	0.388	27.06
**r46**	2.38	0.30	-47.91	-1.99	21.06	0.300	9.92
**r47**	2.40	0.29	-35.44	12.29	25.69	0.479	23.75
**r48**	2.43	0.27	-48.91	-4.54	19.14	0.678	9.09
**r49**	2.45	0.26	-37.77	23.67	26.93	0.377	24.62
**r50**	2.49	0.25	-42.68	28.40	23.64	0.482	22.29
**r51**	2.49	0.25	-37.18	-7.93	19.14	0.407	8.56
Exp.	-	-	67.09	-4.03	22.46	1.000	0.00

[a] Characteristics for representative structure $\boldsymbol{r}_k$ of obtained from the Bim conformational sampling using the AMBER ff14SB forcefield with TIP3P waters. Shown are, the relative cluster free energy (CFE) value in kcal/mol of the corresponding cluster k, the fraction of the ensemble population corresponding to the cluster in percentage, the first two principal components (PC1, PC2) in Fig. S2A, the fraction of the relative accessible surface area (RASA) of the peptide, the R(native)-value and the RMSD in Å of the heavy peptide atoms with respect to the experimental structure. PCA was performed on the distance matrix between the Cα atoms of Bim, excluding i±2 residues using data from both force fields. Subsequently, K-means clustering (k=1000) was performed on the PC coordinates (PC1-PC32) for this force field, followed by R-value scoring of the representative structures from the K-means clusters, where finally the clusters and their representative structures with a cluster free energy (CFE) of 2.5 kcal/mol were retained.

Table S2. Results from Bim sampling simulations (in isolation) with the AMBER ff99SB-ILDN force field with TIP3P waters. [a]

struc. id	Cluster free energy	Population %	PC1	PC2	RASA	R(native)-value	RMSD
**r1**	0.00	8.32	-74.75	2.65	23.73	0.223	20.55
**r2**	0.04	7.78	-14.56	-45.11	30.52	0.148	30.87
**r3**	0.35	4.61	15.13	-73.62	25.58	0.216	20.50
**r4**	0.47	3.79	-46.47	22.31	21.23	0.335	8.23
**r5**	0.48	3.70	-67.34	13.27	19.92	0.118	11.53
**r6**	0.48	3.69	-69.92	-14.36	19.90	0.122	11.68
**r7**	0.62	2.95	-36.70	69.08	34.79	0.107	33.59
**r8**	0.67	2.72	-57.30	-40.95	27.32	0.030	32.12
**r9**	0.69	2.60	-57.73	-19.60	27.12	0.132	25.89
**r10**	0.72	2.47	-24.19	20.46	27.82	0.342	26.32
**r11**	0.76	2.32	-17.13	-43.92	37.01	0.186	33.64
**r12**	0.78	2.26	-41.11	-7.61	18.46	0.166	8.49
**r13**	0.90	1.83	-45.33	46.00	30.57	0.341	29.16
**r14**	0.95	1.68	-23.03	-20.71	20.32	0.177	8.14
**r15**	0.99	1.57	-24.81	34.60	31.72	0.231	27.00
**r16**	1.09	1.34	-16.19	-52.54	29.82	0.151	30.71
**r17**	1.13	1.25	-46.75	-14.88	19.79	0.171	8.26
**r18**	1.19	1.13	-24.60	-22.29	20.80	0.137	7.68
**r19**	1.26	1.00	-45.36	-5.12	29.39	0.127	33.43
**r20**	1.31	0.92	0.41	-35.94	29.69	0.223	26.49
**r21**	1.39	0.80	-66.10	1.32	26.24	0.110	23.94
**r22**	1.40	0.80	-83.22	-8.49	29.04	0.116	29.79
**r23**	1.49	0.68	-44.84	34.45	21.74	0.304	14.54
**r24**	1.51	0.66	-34.87	-23.51	24.32	0.316	20.21
**r25**	1.54	0.63	-38.26	-16.71	20.63	0.122	8.47
**r26**	1.55	0.62	-32.62	17.86	25.60	0.424	22.55
**r27**	1.56	0.61	-63.28	12.41	23.80	0.510	17.77
**r28**	1.57	0.60	-44.21	-19.69	19.67	0.147	8.31
**r29**	1.58	0.59	-55.39	9.08	18.40	0.157	9.08
**r30**	1.59	0.58	-55.44	-44.35	23.31	0.274	15.66
**r31**	1.63	0.54	-46.47	-3.92	21.65	0.164	8.92
**r32**	1.65	0.52	-47.96	8.53	26.34	0.438	22.32
**r33**	1.65	0.52	-70.40	8.40	19.74	0.251	9.89
**r34**	1.68	0.49	-49.84	-14.69	32.43	0.375	29.63
**r35**	1.68	0.49	-50.78	32.40	19.51	0.279	8.79
**r36**	1.70	0.48	-44.75	10.34	30.79	0.218	32.44
**r37**	1.73	0.46	-39.10	7.68	20.68	0.355	8.60
**r38**	1.74	0.45	-52.89	1.37	20.74	0.105	10.15

**r39**	1.76	0.44	-59.81	-0.64	27.63	0.369	28.01
**r40**	1.81	0.40	-70.66	22.66	28.15	0.061	29.69
**r41**	1.81	0.40	-49.62	8.76	27.42	0.259	25.67
**r42**	1.82	0.39	-8.21	75.70	23.19	0.075	9.11
**r43**	1.84	0.38	-48.84	20.29	25.78	0.337	30.42
**r44**	1.85	0.37	-83.03	3.17	27.77	0.071	28.72
**r45**	1.86	0.37	-48.95	19.49	24.87	0.673	21.01
**r46**	1.87	0.36	-67.77	-8.51	31.71	0.385	31.61
**r47**	1.87	0.36	-72.19	9.59	21.34	0.266	12.74
**r48**	1.88	0.35	-14.21	-69.73	20.99	0.162	9.02
**r49**	1.95	0.32	-62.49	-8.88	24.86	0.408	19.00
**r50**	1.95	0.31	-51.91	18.21	22.84	0.202	17.09
**r51**	1.96	0.31	14.20	-25.76	23.83	0.544	18.96
**r52**	1.97	0.30	-39.72	61.82	32.54	0.196	32.31
**r53**	1.98	0.30	-49.82	-21.25	29.05	0.132	28.78
**r54**	1.98	0.30	-37.76	-41.94	29.16	0.124	28.95
**r55**	1.99	0.29	-36.10	46.47	26.30	0.235	24.68
**r56**	2.03	0.28	-56.58	4.43	31.81	0.275	33.15
**r57**	2.04	0.27	5.15	82.02	24.13	0.043	18.26
**r58**	2.06	0.26	-46.87	9.85	35.41	0.261	32.25
**r59**	2.07	0.26	-6.20	-37.95	19.22	0.340	8.28
**r60**	2.07	0.26	-54.01	-27.30	34.83	0.255	36.47
**r61**	2.08	0.26	-55.41	-25.35	19.55	0.313	8.76
**r62**	2.08	0.25	-61.83	-25.23	20.89	0.183	11.06
**r63**	2.11	0.24	-36.33	7.46	20.47	0.564	9.98
**r64**	2.11	0.24	-19.26	-30.37	25.00	0.231	17.81
**r65**	2.12	0.24	-27.19	22.21	26.87	0.261	25.30
**r66**	2.12	0.24	-53.81	-0.71	20.70	0.207	8.94
**r67**	2.15	0.22	-60.31	5.74	25.31	0.105	26.97
**r68**	2.15	0.22	-33.82	25.21	22.83	0.611	13.74
**r69**	2.16	0.22	-53.32	-16.54	19.71	0.560	8.87
**r70**	2.16	0.22	-37.79	-28.56	20.50	0.130	9.16
**r71**	2.19	0.21	6.73	-20.13	20.43	0.400	7.02
**r72**	2.20	0.21	-20.56	-42.25	27.08	0.337	23.39
**r73**	2.20	0.21	2.05	-3.99	24.91	0.399	20.03
**r74**	2.21	0.20	-66.10	19.49	29.23	0.069	29.28
**r75**	2.22	0.20	-67.42	-0.02	21.80	0.265	17.84
**r76**	2.23	0.20	-42.18	-1.11	29.35	0.045	30.50
**r77**	2.23	0.20	-41.54	-0.87	22.37	0.279	16.41
**r78**	2.25	0.19	-11.60	40.51	33.86	0.260	31.81
**r79**	2.25	0.19	-66.61	21.97	19.84	0.288	9.79
**r80**	2.26	0.19	-73.11	16.98	21.47	0.078	13.64
**r81**	2.27	0.19	-34.80	29.47	20.72	0.234	8.85

**r82**	2.28	0.18	-48.59	42.71	22.03	0.200	18.23
**r83**	2.29	0.18	-50.51	20.44	19.50	0.249	8.91
**r84**	2.29	0.18	-23.59	45.99	23.71	0.241	9.61
**r85**	2.29	0.18	-35.16	42.09	25.43	0.454	26.30
**r86**	2.29	0.18	-42.85	-8.22	22.98	0.306	18.62
**r87**	2.31	0.17	-46.45	-22.26	28.18	0.373	27.34
**r88**	2.31	0.17	-37.37	-12.03	26.34	0.315	19.06
**r89**	2.32	0.17	-35.16	-1.70	25.26	0.520	24.30
**r90**	2.33	0.17	-19.43	-18.97	30.35	0.253	28.16
**r91**	2.34	0.16	-35.42	11.98	25.80	0.465	26.54
**r92**	2.34	0.16	-3.04	-16.96	27.03	0.417	20.70
**r93**	2.35	0.16	-37.07	11.94	26.41	0.410	26.50
**r94**	2.35	0.16	-22.36	60.33	26.30	0.117	18.69
**r95**	2.37	0.15	-68.70	-15.75	20.86	0.096	10.66
**r96**	2.38	0.15	31.60	26.87	30.16	0.511	23.76
**r97**	2.39	0.15	-44.34	33.98	32.51	0.312	30.49
**r98**	2.39	0.15	-20.56	8.71	23.52	0.489	17.02
**r99**	2.40	0.15	-44.71	15.87	24.79	0.099	17.17
**r100**	2.41	0.14	-64.74	35.28	28.72	0.316	28.00
**r101**	2.42	0.14	-79.01	0.51	18.76	0.500	9.47
**r102**	2.43	0.14	-40.84	22.43	22.65	0.244	11.82
**r103**	2.43	0.14	-51.57	-7.36	21.78	0.173	12.96
**r104**	2.44	0.14	-23.59	61.30	27.71	0.130	20.00
**r105**	2.45	0.14	-45.18	39.92	27.23	0.316	31.91
**r106**	2.45	0.14	-31.59	-21.04	20.05	0.281	8.29
**r107**	2.46	0.14	-34.95	-7.06	31.40	0.370	32.96
**r108**	2.46	0.13	-71.58	1.44	25.04	0.187	20.69
**r109**	2.47	0.13	-19.26	11.26	18.77	0.717	6.18
**r110**	2.48	0.13	-37.41	-21.46	22.34	0.239	8.97
**r111**	2.48	0.13	-0.98	-42.48	26.27	0.068	21.96
**r112**	2.49	0.13	-57.11	23.03	23.83	0.251	19.02
**Exp.**	-	-	55.25	-5.15	22.44	1.00	0.00

[a] Characteristics for representative structure $\boldsymbol{r}_k$ of obtained from the Bim conformational sampling using the AMBER ff99SB-ILDN forcefield with TIP3P waters. Shown are, the relative cluster free energy (CFE) value in kcal/mol of the corresponding cluster *k*, the fraction of the ensemble population corresponding to the cluster in percentage, the first two principal components (PC1, PC2) in Fig. S2B, the fraction of the relative accessible surface area (RASA) of the peptide, the R(native)-value and the RMSD in Å of the heavy peptide atoms with respect to the experimental structure.

PCA was performed on the distance matrix between the Cα atoms of Bim, excluding i±2 residues using data from both force fields. Subsequently, K-means clustering (k=1000) was performed on the PC coordinates (PC1-PC31) for this force field, followed by R-value scoring of the representative structures from the K-means clusters, where finally the clusters and their representative structures with a cluster free energy (CFE) of 2.5 kcal/mol were retained.

Table S3. Convergence of McMD dynamic docking simulations. [a]

Iteration	Multicanonical coverage (%)	Standard deviation	Simulation length (ns)
0	6.3	-	1.0
1	14.1	3.431	0.1
2	23.1	2.221	0.2
3	33.9	3.030	0.3
4	48.7	1.831	0.4
5	61.1	1.656	0.5
6	71.2	1.703	0.5
7	79.3	1.615	0.6
8	87.3	1.061	0.7
9	94.2	0.948	0.8
10	100.0	1.031	0.9
11	100.0	0.535	1.0
12	100.0	0.373	1.0
13	100.0	0.194	2.0
14	100.0	0.236	2.0
15	100.0	0.324	3.0
16	100.0	0.351	3.0
17	100.0	0.368	4.0
18	100.0	0.857	4.0
19	100.0	0.800	4.0
20	100.0	0.947	8.0
21	100.0	0.936	8.0
22	100.0	1.280	8.0
23	100.0	0.785	8.0
24	100.0	0.672	12.0
25	100.0	0.380	12.0
26	100.0	0.167	12.0
27	100.0	0.320	12.0
28	100.0	0.374	16.0
29	100.0	0.168	16.0
30	100.0	0.333	16.0
31	100.0	0.340	16.0
32	100.0	0.291	18.0
33	100.0	0.331	32.0
34	100.0	0.348	32.0
35	100.0	0.317	32.0
36	100.0	0.443	32.0
37	100.0	0.491	32.0
38	100.0	0.276	32.0

39	100.0	0.261	32.0
40	100.0	0.129	32.0
41	100.0	0.139	32.0
42	100.0	0.223	32.0
43	100.0	0.260	32.0
44	100.0	0.251	32.0
45	100.0	0.219	32.0
46	100.0	0.281	32.0
47	100.0	0.187	32.0
48	100.0	0.183	64.0
49	100.0	0.128	64.0

[a] Overview of McMD pre-run, with for each iteration, the multicanonical coverage (i.e., temperature coverage) in percentage (with respect to the full 280 K – 700 K range), flatness of the multicanonical potential energy distribution measured as the standard deviation of the log-probability values corresponding to the energies within the multicanonical range and the simulation length per trajectory (N=30).

Table S4. McMD-based dynamic docking results using subsets of the simulation data[a]

25 % (7.5 μs)					
	**CFE (kcal/mol)**	**RASA**	**R(native)-value**	**RMSD (Å)**	**λ (Å)**
$\mathbf{r}_1$	0.00	0.51	0.674	2.72	5.27
$\mathbf{r}_2$	0.48	0.62	0.294	6.86	7.28
$\mathbf{r}_3$	0.63	0.57	0.407	6.29	7.73
$\mathbf{r}_4$	0.65	0.63	0.134	12.50	10.33
$\mathbf{r}_5$	1.02	0.54	0.634	5.73	4.55
$\mathbf{r}_6$	1.15	0.54	0.028	16.21	7.34
$\mathbf{r}_7$	1.34	0.60	0.572	5.88	4.37
$\mathbf{r}_8$	1.43	0.63	0.098	9.24	9.27
$\mathbf{r}_9$	1.44	0.57	0.625	4.69	4.99
$\mathbf{r}_{10}$	2.05	0.57	0.613	5.99	4.73
$\mathbf{r}_{11}$	2.16	0.54	0.063	16.65	6.23
$\mathbf{r}_{12}$	2.36	0.61	0.549	5.64	7.82
$\mathbf{r}_{13}$	2.37	0.62	0.366	8.09	7.01
Exp	-	0.53	1.000	0.00	5.94

50 % (15 μs)					
	**CFE (kcal/mol)**	**RASA**	**R(native)-value**	**RMSD (Å)**	**λ (Å)**
$\mathbf{r}_1$	0.00	0.52	0.711	2.67	5.88
$\mathbf{r}_2$	0.61	0.53	0.625	4.46	4.48
$\mathbf{r}_3$	0.63	0.59	0.300	6.90	7.92
$\mathbf{r}_4$	0.82	0.62	0.141	12.64	10.75
$\mathbf{r}_5$	1.21	0.57	0.407	6.29	7.73
$\mathbf{r}_6$	1.28	0.54	0.030	16.16	7.33
$\mathbf{r}_7$	1.40	0.53	0.569	8.44	7.47
$\mathbf{r}_8$	1.84	0.55	0.617	6.05	4.25
$\mathbf{r}_9$	2.00	0.63	0.098	9.24	9.27
$\mathbf{r}_{10}$	2.12	0.66	0.126	14.35	8.97
$\mathbf{r}_{11}$	2.15	0.64	0.094	17.18	10.92
$\mathbf{r}_{12}$	2.22	0.61	0.391	8.16	6.17
$\mathbf{r}_{13}$	2.30	0.60	0.113	13.16	10.46
Exp	-	0.53	1.000	0.00	5.94

75 % (22.5 μs)					
	**CFE (kcal/mol)**	**RASA**	**R(native)-value**	**RMSD (Å)**	**λ (Å)**
$\mathbf{r}_1$	0.00	0.54	0.715	3.21	5.42
$\mathbf{r}_2$	0.32	0.54	0.634	3.21	5.30
$\mathbf{r}_3$	0.67	0.63	0.294	6.80	7.46

$\mathbf{r}_4$	0.82	0.65	0.127	12.69	10.8
$\mathbf{r}_5$	1.00	0.52	0.566	8.13	7.52
$\mathbf{r}_6$	1.26	0.51	0.022	16.18	7.40
$\mathbf{r}_7$	1.51	0.56	0.424	6.52	7.75
$\mathbf{r}_8$	1.77	0.59	0.609	6.09	4.19
$\mathbf{r}_9$	1.96	0.68	0.109	17.24	9.97
$\mathbf{r}_{10}$	2.02	0.56	0.680	3.45	5.82
$\mathbf{r}_{11}$	2.23	0.56	0.464	7.04	6.92
$\mathbf{r}_{12}$	2.30	0.63	0.098	9.24	9.27
$\mathbf{r}_{13}$	2.42	0.66	0.126	14.35	8.97
$\mathbf{r}_{14}$	2.45	0.64	0.100	17.45	10.85
$\mathbf{r}_{15}$	2.46	0.51	0.524	3.96	5.90
Exp	-	0.53	1.000	0.00	5.94

[a] The McMD-based dynamic docking ensemble was re-analyzed using subsets of the simulation data, starting from the PCA to the analysis of the top ranked (less than 2.5 kcal/mol) structures $\mathbf{r}_k$, with the statistics of those structures shown in the table. E.g., for the 25 % (7.5 µs) dataset, 250 ns of each trajectory (30 parallel trajectories) was used for the PCA, after which those snapshots were re-clustered using those PC coordinates, to finally obtain the representative structures.

Table S5. Per-residue R-values of Bim during 400 K canonical simulations. [a]

	r_1	r_2	r_3	r_4	r_5	r_6	r_7	r_8	r_9
**Ile56b**	0.668 (0.161)	0.435 (0.307)	0.689 (0.219)	0.565 (0.304)	0.275 (0.323)	0.855 (0.196)	0.200 (0.220)	0.511 (0.318)	0.288 (0.431)
**Trp57b**	0.542 (0.249)	0.768 (0.327)	0.768 (0.219)	0.663 (0.339)	0.308 (0.227)	0.949 (0.058)	0.207 (0.241)	0.513 (0.306)	0.748 (0.317)
**Ile58b**	0.707 (0.181)	0.710 (0.336)	0.777 (0.254)	0.000 (0.000)	0.573 (0.236)	0.848 (0.105)	0.499 (0.230)	0.716 (0.420)	0.704 (0.235)
**Ala59b**	0.870 (0.066)	0.832 (0.276)	0.806 (0.221)	0.000 (0.000)	0.899 (0.177)	0.927 (0.095)	0.658 (0.281)	0.528 (0.321)	0.691 (0.268)
**Gln60b**	0.930 (0.081)	0.584 (0.233)	0.883 (0.196)	0.580 (0.314)	0.936 (0.099)	0.367 (0.267)	0.303 (0.291)	0.550 (0.376)	0.000 (0.000)
**Glu61b**	0.927 (0.074)	0.534 (0.214)	0.653 (0.160)	0.861 (0.284)	0.599 (0.114)	0.936 (0.075)	0.210 (0.334)	0.623 (0.230)	0.355 (0.445)
**Leu62b**	0.759 (0.106)	0.784 (0.240)	0.859 (0.083)	0.824 (0.232)	0.813 (0.109)	0.806 (0.345)	0.000 (0.000)	0.719 (0.183)	0.875 (0.256)
**Arg63b**	0.844 (0.099)	0.763 (0.264)	0.606 (0.341)	0.888 (0.191)	0.893 (0.096)	0.581 (0.323)	0.368 (0.252)	0.855 (0.099)	0.840 (0.134)
**Arg64b**	0.985 (0.026)	0.564 (0.200)	0.671 (0.260)	0.580 (0.275)	0.867 (0.112)	0.694 (0.336)	0.535 (0.169)	0.307 (0.234)	0.704 (0.249)
**Ile65b**	0.943 (0.056)	0.505 (0.372)	0.871 (0.101)	0.800 (0.257)	0.895 (0.108)	0.648 (0.297)	0.649 (0.169)	0.825 (0.117)	0.715 (0.181)
**Gly66b**	0.987 (0.010)	0.578 (0.396)	0.978 (0.030)	0.735 (0.250)	0.992 (0.012)	0.919 (0.167)	0.910 (0.077)	0.991 (0.014)	0.679 (0.264)
**Asp67b**	0.965 (0.035)	0.483 (0.485)	0.977 (0.049)	0.599 (0.317)	0.984 (0.021)	0.699 (0.297)	0.872 (0.134)	0.988 (0.019)	0.386 (0.286)
**Glu68b**	0.778 (0.148)	0.368 (0.400)	0.711 (0.210)	0.663 (0.342)	0.686 (0.177)	0.710 (0.298)	0.534 (0.194)	0.718 (0.204)	0.316 (0.333)
**Phe69b**	0.872 (0.088)	0.370 (0.331)	0.830 (0.097)	0.734 (0.315)	0.840 (0.116)	0.833 (0.108)	0.695 (0.262)	0.859 (0.096)	0.491 (0.374)
**Asn70b**	0.976 (0.040)	0.508 (0.346)	0.932 (0.161)	0.767 (0.378)	0.979 (0.044)	0.895 (0.117)	0.729 (0.339)	0.978 (0.052)	0.386 (0.350)
**Ala71b**	0.862 (0.265)	0.523 (0.380)	0.933 (0.211)	0.730 (0.413)	0.388 (0.353)	0.814 (0.158)	0.559 (0.435)	0.507 (0.381)	0.352 (0.326)
**Tyr72b**	0.481 (0.348)	0.477 (0.311)	0.681 (0.287)	0.692 (0.382)	0.156 (0.239)	0.745 (0.204)	0.367 (0.348)	0.399 (0.365)	0.234 (0.219)
**Tyr73b**	0.402 (0.307)	0.299 (0.301)	0.493 (0.268)	0.616 (0.332)	0.409 (0.414)	0.883 (0.087)	0.390 (0.337)	0.362 (0.342)	0.135 (0.233)

[a] Average R-values with standard deviations measured from the final 40 ns of the canonical MD simulations at 400 K for each individual residue of Bim.

Table S6. Picking statistics from the McMD ensemble to produce the binding pathway for the path sampling simulations. [a]

Window λ (Å)	Previous window's structure -> current window's structure R-value (RMSD in Å)	Cutoff	N
1	0.928 (2.83)	R:0.90	6947
2	0.820 (2.26)	R:0.81	36
3	0.750 (7.23)	R:0.74	33
4	0.828 (3.60)	R:0.81	35
5	0.813 (4.75)	R:0.75	30
6	0.544 (5.56)	R:0.7 X:5.0	1
7	0.995 (4.34)	R:0.90	63
8	0.987 (5.22)	R:0.7 X:5.0	9
10	0.386 (7.44)	R:0.7 X:5.0	1
12	0.719 (4.93)	R:0.7 X:5.0	1
14	0.240 (8.32)	R:0.7 X:5.0	1
16	0.745 (5.43)	R:0.7 X:5.0	1
18	0.935 (3.29)	R:0.7 X:5.0	1
20	0.052 (10.30)	R:0.7 X:5.0	1
24	0.447 (7.63)	R:0.0 X:5.0	1
28	0.000 (8.39)	R:0.0 X:5.0	1
32	0.950 (5.08)	R:0.0 X:5.0	1
36	0.223 (10.74)	R:0.0 X:5.0	1

[a] Listed are the statistics for the pathway starting from $\boldsymbol{q_1}$. The algorithm starts at window where λ = 0 Å with the structure $\boldsymbol{q_k}$, and then moves to window +1, picking one representative structure for this window that is similar to $\boldsymbol{q_k}$. Then, the process is repeated for the window +2, picking a structure similar to that of the preceding window, i.e. those from +1. Shown are the similarities between the picked structure from the preceding window to the current window, in terms of the R-value and the RMSD of the ligand. Finally, the cutoff used is listed, where "R:" corresponds to the R-value cutoff and "X:" to the RMSD based cutoff, where matching structures only have to fulfill one of the criteria and the number of matching structures is listed in the final column "N". In case the number of contacts is less than 25, the R-value cutoff is no longer used (in these cases, R:0.0 is set)

Table S7. Distance between Cα atoms of residue pairs in structures $\mathbf{r}_k$ and $\boldsymbol{r}_k^{bcl}$.[a]

	***d*0 (Leu112 - Ser122)**	***d*1 (Leu108 - Val126)**	***d*2 (Phe105 - Leu130)**	***d*3 (Tyr101 - Arg139)**	***d*4 (Glu96 - Asn136)**
$\mathbf{r}_1$	6.40	9.22	12.32	13.30	17.41
$\mathbf{r}_2$	6.90	8.20	10.87	11.89	16.40
$\mathbf{r}_3$	9.39	10.55	12.37	13.66	17.03
$\mathbf{r}_4$	7.20	6.45	10.31	12.94	16.16
$\mathbf{r}_5$	7.35	7.87	11.63	13.76	16.80
$\mathbf{r}_6$	6.40	7.25	10.66	14.52	17.01
$\mathbf{r}_7$	6.44	6.92	10.98	13.22	16.22
$\mathbf{r}_8$	6.34	11.19	13.57	13.32	17.12
$\mathbf{r}_9$	7.61	6.47	10.95	13.22	15.41
$\boldsymbol{r}_1^{bcl}$	7.04	8.27	11.15	12.10	16.24
$\boldsymbol{r}_2^{bcl}$	8.34	12.61	12.92	12.96	16.87
$\boldsymbol{r}_3^{bcl}$	8.89	10.85	15.56	12.00	16.33
$\boldsymbol{r}_4^{bcl}$	7.63	12.00	16.27	13.12	17.09
$\boldsymbol{r}_5^{bcl}$	6.84	7.16	11.30	12.63	17.20

[a] Distance between the Cα atoms are in Å, with the distributions of the full reweighted (300 K) ensemble shown in Fig. S9.

Table S8. Results from Bim sampling simulations (in isolation) with the AMBER ff99SB-ILDN force field and the OPC water force field. [a]

struc. id	Cluster free energy	Population %	PC1	PC2	RASA	R(native)-value	RMSD
r1	0	7.33	-61.65	-16.85	19.91	0.174	8.48
r2	0.14	5.81	-18.46	70.96	22.14	0.059	8.89
r3	0.14	5.79	-17.31	-28.21	21.07	0.181	7.8
r4	0.24	4.89	-57.52	-13.05	20.15	0.177	8.02
r5	0.24	4.87	-44.28	12.99	20.43	0.41	8.81
r6	0.43	3.55	7.97	-66.73	23.86	0.104	8.01
r7	0.61	2.65	-63.99	4.58	18.29	0.18	8.99
r8	0.65	2.46	-32.65	46.88	20.1	0.274	8.32
r9	0.66	2.42	-16.45	-58.16	21.82	0.124	8.79
r10	0.94	1.53	-10.48	-10.5	21.33	0.187	8.09
r11	0.95	1.5	-62.79	-4.49	21.08	0.082	9.98
r12	0.99	1.4	-18.06	91.28	20.91	0.278	9.27
r13	1.15	1.06	-48.88	-22.71	19.57	0.374	8.65
r14	1.23	0.94	-12.91	98.54	22.99	0.053	10.07
r15	1.23	0.93	-20.92	29.06	21	0.476	7.87
r16	1.35	0.77	-32.54	-23.13	23.47	0.094	8.09
r17	1.37	0.74	-40.29	30.51	22.31	0.215	8
r18	1.39	0.71	-54.73	2.11	22.21	0.054	10.67
r19	1.41	0.69	-47.47	-28.31	20.18	0.321	8.36
r20	1.41	0.69	-55.98	15.38	19.9	0.205	9.52
r21	1.42	0.68	-53.86	-3.52	20.03	0.386	8.82
r22	1.43	0.66	-52.01	49.95	21.66	0.109	10.43
r23	1.48	0.61	-42.61	42.15	22.53	0.169	8.31
r24	1.48	0.61	-1.87	16.1	23.42	0.279	7.07
r25	1.49	0.61	-43.97	-50.35	20.71	0.188	9.79
r26	1.5	0.59	-63.97	-5.5	19.35	0.165	9.79
r27	1.5	0.59	-59.8	-8.83	19.88	0.424	9.53
r28	1.51	0.58	6.47	-56.33	23.22	0.139	7.43
r29	1.51	0.58	-13.08	-30.46	21.9	0.184	7.68
r30	1.51	0.58	-65.78	-11.19	21.37	0.037	9.57
r31	1.52	0.57	-52.2	-5.81	20.47	0.36	8.07
r32	1.57	0.53	-62.6	4.02	21.6	0.155	9.11
r33	1.58	0.52	9.19	76.73	23.16	0.089	7.92
r34	1.59	0.51	-48.08	-41.71	21.99	0.142	9.59
r35	1.6	0.5	-20.53	41.86	21.74	0.163	8.35
r36	1.63	0.48	-19.25	23.11	23.56	0.159	8.64
r37	1.65	0.46	26.16	-14.33	22.18	0.553	6.22
r38	1.66	0.45	67.11	-50.47	26.21	0.142	7.63

r39	1.7	0.43	-36.02	46.5	21.91	0.202	8.67
r40	1.7	0.43	-33.85	-71.44	22.53	0.075	10.66
r41	1.73	0.4	-6.87	-24.62	22.77	0.39	8.47
r42	1.74	0.4	-8.61	38.38	25.06	0.08	9.09
r43	1.75	0.39	-13.94	54.26	24.88	0.065	10.27
r44	1.75	0.39	-55.21	-17.74	18.82	0.249	8.35
r45	1.79	0.36	-32.54	16.21	23.44	0.166	9.04
r46	1.79	0.36	95.41	90.7	24.9	0.102	7.53
r47	1.8	0.36	-42.5	-45.2	22.01	0.209	9.34
r48	1.81	0.35	-57.89	20.38	22.59	0.11	9.96
r49	1.81	0.35	-33.68	31.44	22.5	0.163	9.24
r50	1.83	0.34	-21.83	-62.66	21.7	0.158	8.76
r51	1.84	0.34	-21.37	-32.51	22.08	0.195	7.85
r52	1.85	0.33	-42.33	1.11	23.37	0.173	9.6
r53	1.86	0.33	-20.63	-4	21.53	0.161	8.52
r54	1.86	0.32	-28.08	-20.54	21.56	0.125	9.3
r55	1.87	0.32	-9.1	-46.47	22.77	0.129	8.28
r56	1.87	0.32	-56.57	-1	20.97	0.192	9.86
r57	1.87	0.32	-28.71	-40.11	20.83	0.272	8.09
r58	1.87	0.32	-50.17	6.64	23.24	0.057	9.99
r59	1.88	0.32	-57.56	13.33	22.01	0.081	9.57
r60	1.9	0.3	-53.01	-45.24	23.3	0.035	10.67
r61	1.91	0.3	-50.23	-19.11	22.03	0.134	10.18
r62	1.92	0.29	-42.59	38.82	21.3	0.213	9.21
r63	1.92	0.29	-65.18	-6.76	20.11	0.099	8.78
r64	1.93	0.29	-54.04	34.45	21.64	0.166	9.18
r65	1.93	0.29	-36.11	-26.86	20.8	0.18	8.61
r66	1.94	0.28	39.04	1.18	25.06	0.187	7.42
r67	1.94	0.28	-39.79	58.96	22.31	0.094	10.53
r68	1.95	0.28	14.6	67.48	23.22	0.082	7.14
r69	1.96	0.28	-24.6	35.23	21.23	0.241	7.1
r70	1.96	0.27	-28	-11.83	21.52	0.29	8.95
r71	1.96	0.27	-36.43	-43.9	22.39	0.353	9.42
r72	1.97	0.27	-32.29	-2.77	21.23	0.349	8.39
r73	1.98	0.27	-46.59	28.59	20.25	0.439	9.44
r74	1.99	0.26	6.71	-85.75	24.44	0.104	9.67
r75	2.01	0.25	-67.37	-23.22	19.62	0.207	9.41
r76	2.01	0.25	-20.06	64.81	22.15	0.246	9.71
r77	2.02	0.25	-46.68	6.79	22.4	0.169	10.83
r78	2.02	0.25	-3.75	-39.36	21.84	0.152	8.38
r79	2.04	0.24	-58.85	28	21.28	0.107	10.17
r80	2.05	0.24	-33.01	9.32	23.63	0.107	9.65
r81	2.06	0.23	-65.01	-0.39	19.18	0.128	9.18

r82	2.07	0.23	-49.35	-14.12	21.16	0.214	9.96
r83	2.07	0.23	168.13	-8.42	27.62	0.079	7.9
r84	2.07	0.23	-2.32	-51.37	22.01	0.421	7.17
r85	2.08	0.22	-27.12	-23.51	24.44	0.004	10.79
r86	2.11	0.21	-38.76	-13.3	20.51	0.124	7.9
r87	2.11	0.21	194.94	28.35	29.05	0.032	8.67
r88	2.11	0.21	19.5	-1.94	25.77	0.216	7.42
r89	2.12	0.21	-61.89	-10.6	21.77	0.229	9.43
r90	2.12	0.21	-24.6	57.23	21.29	0.178	9.23
r91	2.14	0.2	-42.21	-8.72	21.51	0.414	8.73
r92	2.15	0.2	-55.71	-24.01	21.64	0.027	10.7
r93	2.15	0.2	75.39	16.82	25.96	0.177	6.79
r94	2.16	0.2	-42.33	43.79	20.56	0.092	9.08
r95	2.18	0.19	-26.62	-34.86	20.46	0.145	7.87
r96	2.18	0.19	-43.87	62.46	22.71	0.087	10.19
r97	2.18	0.19	24.67	-21.84	23.69	0.329	6.53
r98	2.18	0.19	98.89	92.74	26.75	0.039	8.22
r99	2.19	0.18	-57.46	22.73	21.86	0.042	9.24
r100	2.2	0.18	38.57	102.62	23.72	0.082	8.55
r101	2.21	0.18	-9.46	44.62	24.55	0.1	8
r102	2.22	0.18	-72.9	4.73	20.73	0.112	10.36
r103	2.23	0.18	35.28	82.52	23.89	0.045	8.04
r104	2.23	0.18	15.33	15.86	23.04	0.313	6.85
r105	2.23	0.17	149.89	28.04	27.81	0.056	7.09
r106	2.23	0.17	-13.18	45.49	21.48	0.266	9.13
r107	2.23	0.17	-30.92	-29.59	22.05	0.206	8.68
r108	2.24	0.17	-48.15	-11.9	20.56	0.235	9.61
r109	2.24	0.17	0.19	4.28	22.07	0.148	7.5
r110	2.27	0.16	-44.33	-13.4	20.48	0.214	8.84
r111	2.27	0.16	-28.64	-2.28	23.2	0.078	8.51
r112	2.28	0.16	-47.38	5.54	22.19	0.266	8.92
r113	2.29	0.16	33.1	9.78	24.59	0.223	7.36
r114	2.3	0.15	55.72	5.2	24.63	0.298	6.38
r115	2.31	0.15	-28.13	9.11	22.54	0.108	8.53
r116	2.33	0.15	92.14	40.8	25.39	0.34	7.07
r117	2.33	0.15	-48.08	10.12	21.97	0.274	9.96
r118	2.34	0.15	2.03	28.37	23.32	0.071	7.55
r119	2.34	0.14	-5.43	-39.53	22.57	0.172	7.89
r120	2.35	0.14	53.93	87.48	25.19	0.057	8.21
r121	2.35	0.14	1.06	-51.38	23.28	0.208	9.39
r122	2.35	0.14	-54.19	-22.66	21.83	0.165	9.16
r123	2.36	0.14	-46.74	20.26	20.71	0.255	8.25
r124	2.36	0.14	-25.79	11.9	20.36	0.344	8.03

r125	2.36	0.14	-47.59	-1.1	21.36	0.217	10.47
r126	2.36	0.14	-41.48	-12.09	20.76	0.381	8.21
r127	2.37	0.14	-63.29	3.79	19.39	0.07	9.4
r128	2.37	0.14	-27.03	40.43	21.91	0.283	8.28
r129	2.38	0.14	-31.75	45.2	21	0.128	8.84
r130	2.38	0.13	-51.81	-0.18	21.91	0.382	8.38
r131	2.38	0.13	-54.22	-38.53	20.65	0.148	8.92
r132	2.39	0.13	-49.49	7.73	21.1	0.409	9.93
r133	2.39	0.13	-42.38	-37.95	22.62	0.248	10.01
r134	2.39	0.13	-45.65	-13.77	22.15	0.2	9.46
r135	2.39	0.13	129.02	49.03	26.74	0.113	6.92
r136	2.39	0.13	53.54	-2.27	25.31	0.177	7.38
r137	2.4	0.13	-50.18	-36.09	19.86	0.201	9.49
r138	2.4	0.13	-71.73	2.49	19.82	0.429	9.51
r139	2.4	0.13	-58.29	14.39	21.68	0.147	9.38
r140	2.41	0.13	30.37	68.02	26.31	0.131	8.13
r141	2.41	0.13	-41.14	44.59	20.9	0.323	9.11
r142	2.42	0.13	-27.91	30.37	23.31	0.042	9.21
r143	2.42	0.13	30.99	24.73	22.88	0.203	6.78
r144	2.43	0.13	-18.22	84.22	23.63	0.104	10
r145	2.43	0.12	-16.33	-0.5	21.83	0.291	6.94
r146	2.43	0.12	7.98	-75.28	23.99	0.187	8.63
r147	2.43	0.12	-38.34	-37.48	22.1	0.203	9.14
r148	2.45	0.12	54.37	-10.12	22.88	0.273	6.02
r149	2.45	0.12	-5.15	41.72	23.18	0.086	7.78
r150	2.46	0.12	76.62	-90.32	25.13	0.192	8.3
r151	2.47	0.12	-23.95	65.98	24.88	0.007	10.76
r152	2.48	0.11	-19.01	30.35	23.2	0.21	8.7
r153	2.48	0.11	21.16	34.22	24.81	0.136	7.31
r154	2.49	0.11	-43.38	43.48	21.97	0.209	9.87
r155	2.49	0.11	83.12	10.9	27.9	0.097	8.34
r156	2.5	0.11	1.34	37.22	22.49	0.185	8.01
**Exp.**	-	-	52.49	-7.41	22.45	1.000	0.00

[a] Characteristics for representative structure $\boldsymbol{r}_k$ of obtained from the Bim conformational sampling using the AMBER ff99SB-ILDN forcefield with OPC waters. Shown are, the relative cluster free energy (CFE) value in kcal/mol of the corresponding cluster k, the fraction of the ensemble population corresponding to the cluster in percentage, the first two principal components (PC1, PC2) in Fig. S2B, the fraction of the relative accessible surface area (RASA) of the peptide, the R(native)-value and the RMSD in Å of the heavy peptide atoms with respect to the experimental structure.

PCA was performed on the distance matrix between the Cα atoms of Bim, excluding i±2 residues using data from both force fields. Subsequently, K-means clustering (k=1000) was performed on the PC coordinates (PC1-PC28) for this force field, followed by R-value scoring of the representative structures from the K-means clusters, where finally the clusters and their representative structures with a cluster free energy (CFE) of 2.5 kcal/mol were retained.

Table S9. System & simulation parameters for Bcl-xL – Bim binding simulations.

Parameter	Value
**Number of atoms (total)**	42103
**Number of atoms (Bcl-xL)**	2307
**Number of atoms (Bim)**	319
**Number of water molecules**	13141
**Salt concentration**	0.1 M NaCl
**Box size (pre-NPT)**	110.0 x 62.5 x 62.5 Å
**Box size (post-NPT)**	109.4 x 62.2 x 62.2 Å
**Thermostat**	Bussi (V-rescale)
**Barostat (NPT only)**	Bussi (C-rescale)
**Electrostatics**	Zero-Dipole
**Cutoff (LJ & elec)**	12 Å
**Timestep**	2 fs
**Constraints**	LINCS for solute, SETTLE for waters
**McMD parallel trajectories**	30
**McMD temperature range**	280 K – 700 K
**Pre-run simulation time**	0.8 µs per trajectory
**Production-run simulation time**	1.0 µs per trajectory

SUPPLEMENTARY REFERENCES

1. Nakajima, N., Nakamura, H. & Kidera, A. Multicanonical Ensemble Generated by Molecular Dynamics Simulation for Enhanced Conformational Sampling of Peptides. *J. Phys. Chem. B* **101**, 817–824 (1997).
2. Nakajima, N., Higo, J., Kidera, A. & Nakamura, H. Free energy landscapes of peptides by enhanced conformational sampling 1 1Edited by B. Honig. *J. Mol. Biol.* **296**, 197–216 (2000).
3. Higo, J. *et al.* Energy landscape of a peptide consisting of α-helix, 3_{10} -helix, β-turn, β-hairpin, and other disordered conformations. *Protein Sci.* **10**, 1160–1171 (2001).
4. Kamiya, N., Higo, J. & Nakamura, H. Conformational transition states of a β-hairpin peptide between the ordered and disordered conformations in explicit water. *Protein Sci.* **11**, 2297–2307 (2002).
5. Ikeda, K. & Higo, J. Free-energy landscape of a chameleon sequence in explicit water and its inherent α/β bifacial property. *Protein Sci.* **12**, 2542–2548 (2009).
6. Kamiya, N., Yonezawa, Y., Nakamura, H. & Higo, J. Protein-Inhibitor Flexible Docking by a Multicanonical Sampling: Native Complex Structure with the Lowest Free Energy and a Free-Energy Barrier Distinguishing the Native Complex from the Others. *Proteins* **70**, 41–53 (2008).
7. Ikebe, J. *et al.* Theory for Trivial Trajectory Parallelization of Multicanonical Molecular Dynamics and Application to a Polypeptide in Water. *J. Comput. Chem.* **32**, 1286–1297 (2011).
8. Bekker, G.-J. *et al.* Accurate Prediction of Complex Structure and Affinity for a Flexible Protein Receptor and Its Inhibitor. *J. Chem. Theory Comput.* **13**, 2389–2399 (2017).
9. Bekker, G.-J. & Kamiya, N. Dynamic Docking Using Multicanonical Molecular Dynamics: Simulating Complex Formation at the Atomistic Level. in *Protein-Ligand Interactions and Drug Design* (ed. Ballante, F.) vol. 2266 187–202 (Springer US, 2021).

10. Czabotar, P. E., Lessene, G., Strasser, A. & Adams, J. M. Control of apoptosis by the BCL-2 protein family: implications for physiology and therapy. *Nat. Rev. Mol. Cell Biol.* **15**, 49–63 (2014).
